# Supplementary material for: Precision Medicine Study of Post-Exertional Malaise Epigenetic Changes in Myalgic Encephalomyelitis/Chronic Fatigue Patients During Exercise
Source: Int J Mol Sci. 2025 Sep 3;26(17):8563. doi: 10.3390/ijms26178563 (PMC12429597; doi:10.3390/ijms26178563)
Supplement: Supplementary file 1 [file ijms-26-08563-s001.zip › Supplementary Figures.pdf]

Supplementary Figure S1

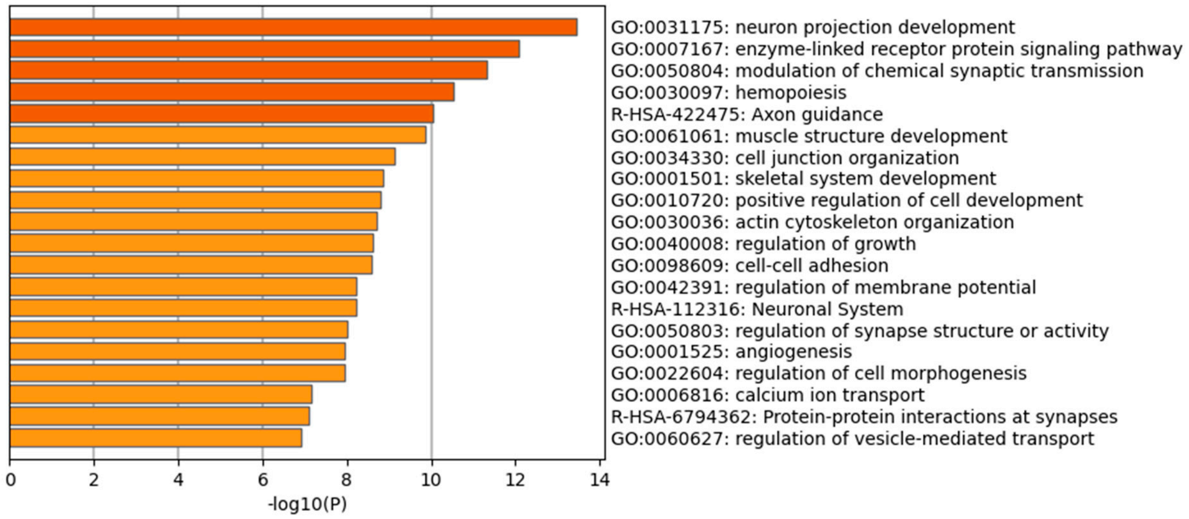

Supplementary Figure 1 Functional pathway enrichment of genes associated with hyper-and hypomethylated DMFs in control. Top GO terms for all unique gene-linked DMFs at promoters and gene bodies in controls

Supplementary Figure S2

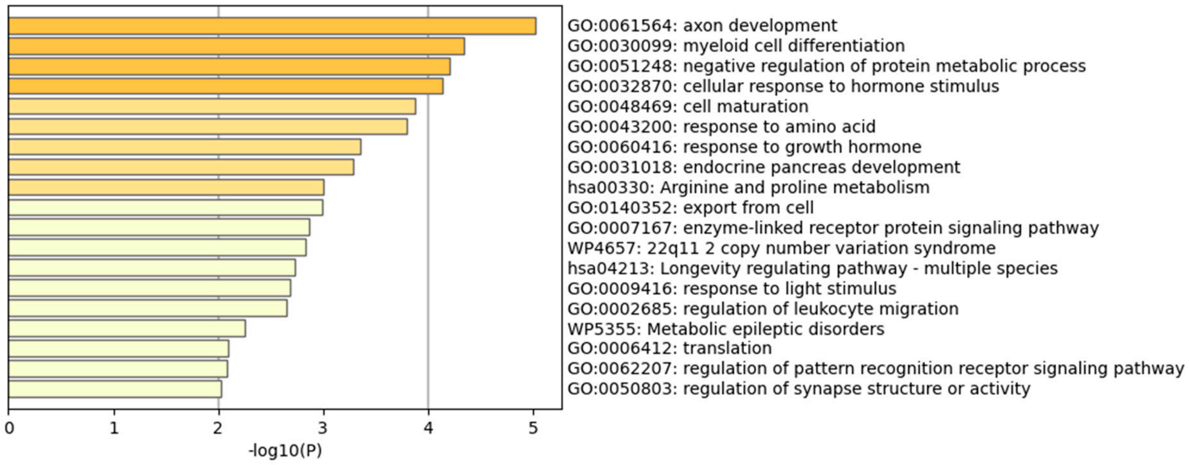

Supplementary Figure 2 Top GO terms all for unique gene-linked DMFs at promoters in Controls

Supplementary Figure S3

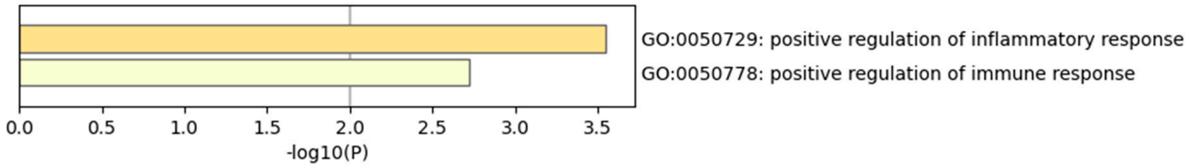

Supplementary Figure 3 Top GO terms all for ME/CFS unique gene-linked DMFs at promoters
